# Differences in corneal clinical findings after standard and accelerated cross-linking in patients with progressive keratoconus

**DOI:** 10.1186/s12886-017-0610-4

**Published:** 2017-11-28

**Authors:** Karsten U. Kortuem, Efstathios Vounotrypidis, Alexandros Athanasiou, Michael Müller, Alexander Babenko, Christoph Kern, Siegfried Priglinger, Wolfgang J. Mayer

**Affiliations:** Department of Ophthalmology, Ludwig-Maximilians-University Munich, Mathildenstr. 8, 80336 Munich, Germany

**Keywords:** Keratoconus, Cross-linking, Accelerated cross-linking, Corneal findings

## Abstract

**Background:**

The purpose of this study was to identify differences in clinical corneal findings after standard and accelerated epithelial off cross-linking (CXL) during a long-term follow-up.

**Methods:**

Two hundred forty-one patients (184 male) were included in this monocentric, retrospective, non-randomized and unmasked study. One hundred forty-eight eyes were treated with the accelerated protocol and 138 with the standard protocol with epithelial off CXL, if diagnosed with keratoconus and a progression in Kmax of more than one dioptre during the preceding 6 months, plus a minimal pachymetry measurement of 400 μm in keratometry (Pentacam, Oculus GmbH, Wetzlar, Germany). Exclusion criteria were previous surgery, other corneal conditions or age above 50 years. Follow-up time was 36 months with clinical examination and keratometry at every visit. Outcome measures were the observed rate of corneal changes, differences between treatment groups and correlation with keratometry measurements.

**Results:**

In patients with accelerated CXL, significantly more clear corneas were seen at three (*p* = 0.015) and six (*p* = 0.002) months after surgery than following the standard protocol. The rate of clear corneas dropped from 52.2% pre-operation (OP) to a minimum of 19.3% after 6 months in the standard protocol group compared with 50.7% clear corneas pre-OP and a minimum of 40.8% in the accelerated group. In the standard protocol group, more striae were found 3 months after intervention than in the accelerated group (*p* = 0.05).

**Conclusions:**

In patients with accelerated CXL, fewer morphological corneal changes were observed than after conventional CXL. However, rarely, corneal changes persisted for a long time.

## Background

Keratoconus is a mostly bilateral non-inflammatory corneal disease that alters stability and refractive power because of progressive thinning and protrusion, mostly in the inferior part of the cornea [1]. More men than women, predominantly in adolescence, are affected [2]. As this is a progressive disease, close monitoring is essential to detect any subtle change in measurement values. In addition to clinical examination, monitoring is achieved in most cases by using a digital keratometric system that measures the anterior and posterior corneal curvature for the detection of early changes [3–5]. A recent study has shown that the number of corneal transplantations can be reduced significantly by the use of a cornea-preserving therapeutically option named corneal cross-linking (CXL) [6].

CXL in patients with keratoconus was first performed in a prospective non-randomized clinical trial in 2003 and aimed at halting the progression of the disease by strengthening the cornea [7]. Success had previously been shown in animal eyes [8]. In this first study in patients, treatment was performed with riboflavin drops and ultraviolet-A (UVA) irradiation (370 nm, 3 mW/cm2 for 30 min), i.e. with the so-called “Dresden protocol”.

Meanwhile, an accelerated protocol was developed, whereby the radiation power was increased, but the duration of the treatment was reduced. This accelerated treatment was recently compared, in humans, with the standard Dresden protocol and proved to be as effective for corneal flattening as the original Dresden protocol [9]. This method is used nowadays routinely in clinics worldwide. Protocols in which the epithelium is not removed, some of them also involving the use of Iontophoresis, have proved to be inferior [10].

Despite CXL being a quicker and less invasive therapy than perforating keratoplasty, significant side effects can arise with regard to this treatment strategy [11]. Usually, in most patients, corneal haze is visible after therapy. Furthermore, some patients suffer from sterile inflammation or scaring that persists and can decrease visual acuity.

The aim of this study has been to analyse the long-term clinical changes in human corneas after CXL carried out because of progressive keratoconus in two observational groups treated at Munich’s University Eye Hospital. One group was treated according to the original Dresden protocol, whereas the second group was treated according to the accelerated protocol. Clinical findings were correlated with various measurements. Clinical and measurement data were retrieved from the Smart Eye Database, which incorporates real-life data from more than 350,000 patients [12].

## Methods

### Patients

Patients were referred from ophthalmological practices, mostly located in the state of Bavaria, Germany, to the University Eye Hospital Munich, because of confirmed or suspected keratoconus, for further evaluation or treatment. Patients were informed about CXL therapy and gave consent to the procedure. Approval for this study was provided by the Institutional Review Board (IRB) of the University Eye Hospital of Munich and adhered to the tenets of the Declaration of Helsinki. Due to the retrospective nature of this study, the IRB ruled, that no consent for participation was necessary. All patients included in this study had progressive keratoconus at least in one eye, defined as an increase in the maximum keratometry (Kmax) value in Pentacam (Oculus GmbH, Wetzlar, Germany) measurements of more than one dioptre in the preceding 6 months. Patients were told not to wear contact lenses 5 days prior to the measurement. A minimal corneal thickness of 400 μm or more and a clear cornea with no scarring was required for treatment inclusion. Exclusion criteria included previous ocular trauma or surgery, corneal disease (other than keratoconus) or systemic disease that might affect the cornea, stable conditions of keratoconus and a study subject age of more than 50 years.

### Surgical treatment

From 2009 to 2013, all patients received treatment according to the Dresden protocol via an UVX-1000 device (IROC; Switzerland, 3 mW, 30 min). All Patients recruited from 2013 to 2016 were treated according to an accelerated protocol by using an UVX-2000 device (IROC; Switzerland, 9 mW, 10 min). In this retrospective analysis, assignment of patients to treatment protocols was only done by the date of CXL and the current standard of care at that time (Until 2013: Dresden protocol, from 2013: accelerated protocol). No further considerations were made. In all cases, CXL was performed as an outpatient procedure under topical anaesthesia by using the “epithelial-off” technique. After epithelial debridement, riboflavin drops were instilled every 5 min for 30 min in the Dresden protocol group (Medio Cross isotone, Peschke GmbH, Waldshut-Tiengen, Germany) and every 2 min for 10 min in the accelerated group (VibeX rapid, Avedro Inc., Waltham, MA, USA). Immediately afterwards, light irradiation was started in the Dresden protocol group for 30 min and in the accelerated group for 10 min. The source was mounted at 5 cm distance from the corneal apex to cover the whole cornea with light irradiation. To reduce pain and irritation, a soft bandage contact lens was inserted after surgery until the epithelium healed. All surgical interventions were uneventful. Patients were treated postoperatively with preservative-free Levofloxacin antibiotic eye drops and dexamethasone eye drops, both 4 times daily. Lubricating eye drops were also prescribed. Treatment with the antibiotic drops was stopped on contact lens removal after four to 5 days. Dexamethasone eye drops were reduced by one drop per week.

### Follow-up period

On every follow-up examination (1–3, 6, 9, 12, 24 and 36 months after the procedure; except for the first post-operative (OP) day), a Pentacam (Oculus, Wetzlar, Germany) examination was undertaken by using the same device. Patients were instructed not to wear contact lenses (if applicable) 5 days prior to every follow-up examination, in order to reduce effects of corneal warpage. As not all patients attended at the same time intervals, the incidence of findings in the intervals 0–3, 3–6, 6–9, 9–12, 12–24 and 24–36 months were assessed. For every clinical examination, the corresponding measurements (closest to examination date) from the Pentacam were included. In cases of differences between the Pentacam measurement and clinic dates, the values were adjusted by linear interpolation. A clinical examination that included an assessment of corneal findings (haze, striae, infiltrates, opacification, scar, oedema and epithelial defect) was performed. Uncorrected and corrected visual acuity was tested.

### Data analysis

Data were exported from the Smart Eye Database, which includes all patient data from the electronic health record and measurement values from the Pentacam for every examination. Statistical analysis was performed by using R software (https://www.r-project.org/, The R project for Statistical Computing Version 3.2.2). Based on the changes of incidences of fixed corneal findings between the pre-OP and post-OP control points, the exact McNemar test was used to detect significant differences within the progress of clinical findings in both groups. The exact McNemar Test was applied to the 2 × 2-matrix consisting of the incidences for finding/finding, finding/no finding, no finding/finding and no finding/no finding at the pe-OP/post-OP-control-point for every finding and every post-OP-control-point. In particular, relationships between clinical findings and Pentacam measurement values were established by using the Mann-Whitney U-test.

Moreover, differences between the treatment protocols were determined by considering the number of incidences for fixed findings and control-points and by applying the exact Fisher test to the related 2 × 2-matrix. To obtain an additional value for dissimilarity beside the results of the McNemar test and the Fisher test, odds ratio values were established. An odds ratio of <1 means a change from “finding occurs” to “finding does not occur”, whereas an odds ratio of >1 implies the opposite conclusion. All results were considered statistically significant at *p* < 0.05.

## Results

### Patients and pre-treatment measurements

For this study, we included 241 patients (184 male patients, average age 27.9 years), with 286 treated eyes. Before treatment, Kmax was 54.25 D and Kmean front (Kmf) was 46.34 D in the accelerated treatment group, whereas in the Dresden protocol treatment group, Kmax was 55.53 D and Kmf 47.76 D. The average pachymetry was 467.62 μm (accelerated) or 457.75 μm (Dresden protocol) pre-operatively. Table 1 shows the baseline data of both groups. A normal distribution of pre-OP Pentacam measurements was seen according to the Anderson-Darling test.

**Table 1:** Patient demographics

Items:	Accelerated:	Dresden protocol:
Number of patients (male)	131 (98)	110 (86)
Eyes (male)	148 (110)	138 (109)
Average age (male/female)	28.7+/−11.6 (27.6/31.9)	27.0+/−9.4 (25.7/ 31.9)
Right eyes (m/f)	68 (45/23)	76 (56/20)
Kmax pre-OP (mean, sd, median)	54.3, 5.6, 53.8	55.5, 5.6, 55.4
Pachmetry pre-OP (mean, sd, median)	467.6, 37.0, 466.5	457.6, 31.6, 460.9
KMF pre-OP (mean, sd, median)	46.3, 3.5, 45.9	47.8, 3.4, 47.1
Visual acuity pre-OP (logmar) (mean, sd, median)	cc: 0.34, 0.24, 0.3 sc: 0.47, 0.33, 0.4	cc: 0.37, 0.28, 0.35 sc: 0.55, 0.37, 0.49

Patient demographics and characteristics. *Kmax* Maximal K value as measured in keratometry, *Kmf* average keratometry reading of front cornea, *cc* cum correctione (with best correction), *sc* sine correctione (without any correction)

### Corneal findings during treatment

**Fig. 1:**
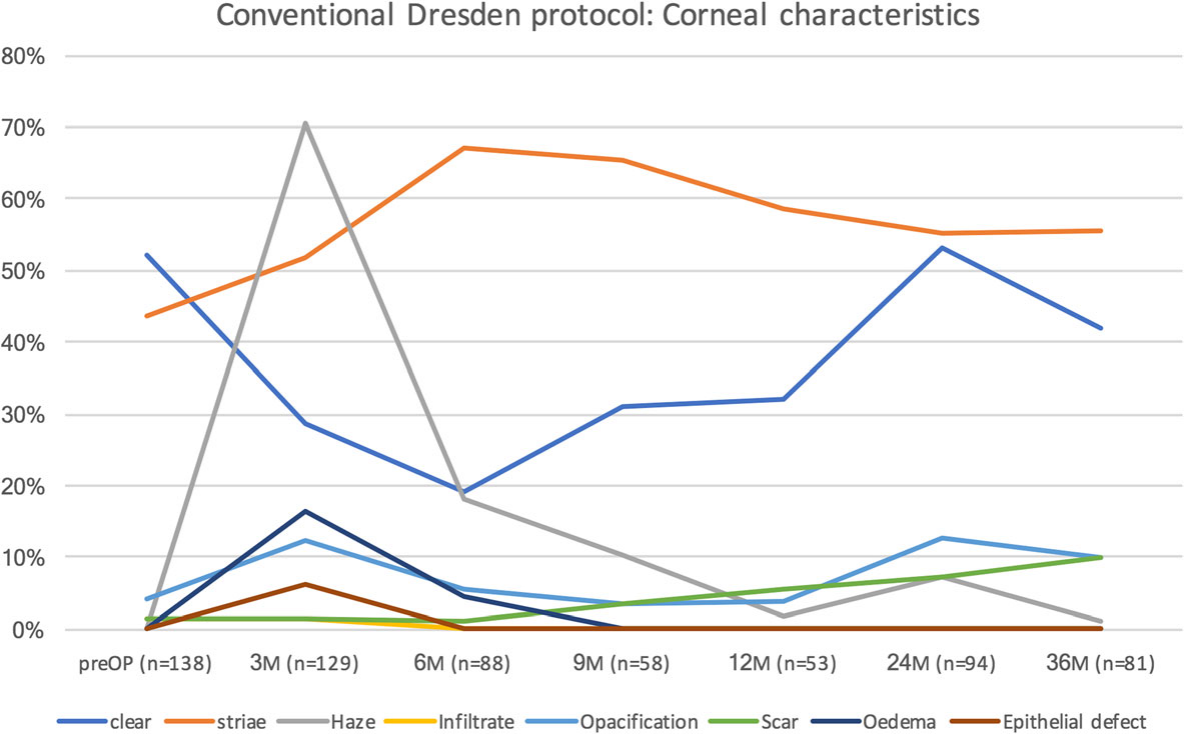
Conventional Dresden protocol: corneal characteristics and their extent during therapy

**Fig. 2:**
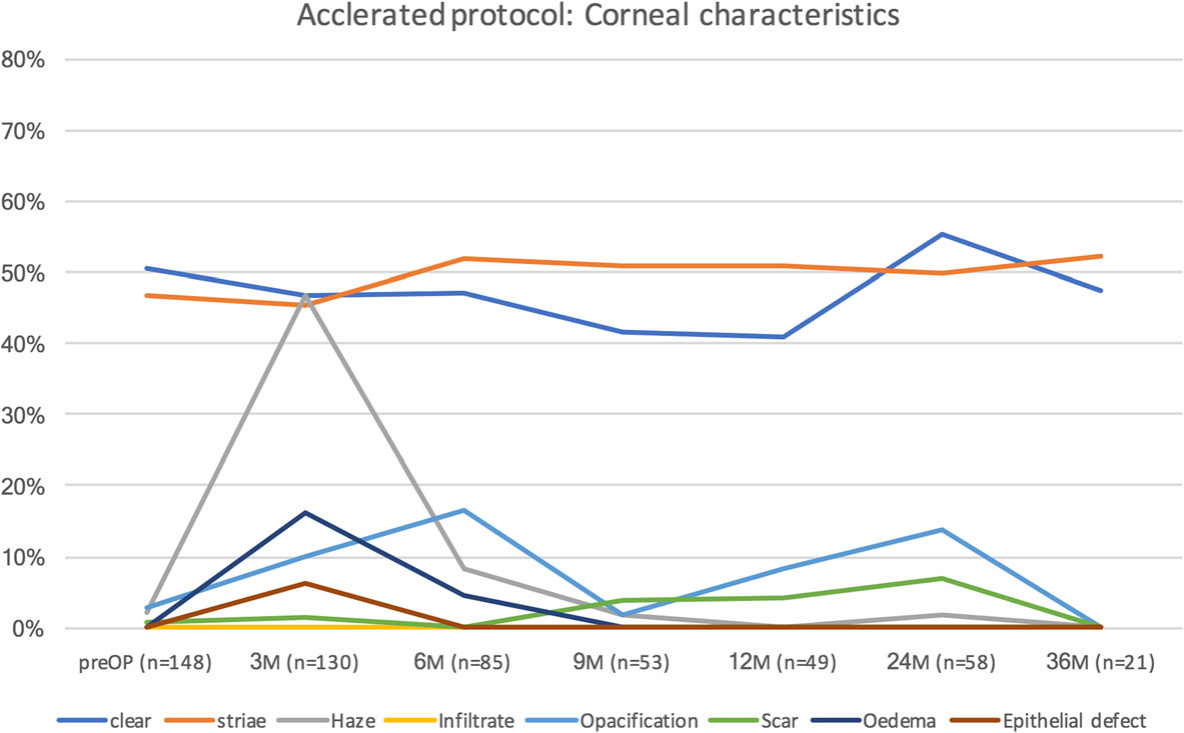
Accelerated treatment protocol: corneal characteristics and their extent during therapy

The course of corneal findings during therapy is shown in Fig. 1 for the conventional Dresden protocol and Fig. 2 for the accelerated treatment. Before treatment, 52.2% (Dresden protocol) or 50.7% (accelerated) of corneas were clear and 43.5% (Dresden) or 46.6% (accelerated) showed striae. The rate of clear corneas dropped during treatment to a minimum of 19.3% after 6 months (Dresden) and to 40.8% after 12 months (accelerated). For striae, the peak was at 67% after 6 months (Dresden) and dropped to 55.7% after 36 months. In the accelerated protocol, the peak of striae findings also occurred after 36 months, but at a value of 52.4% with no intermittent rise. Haze peaked in both protocols after 3 months at 70.5% (Dresden) and at 46.9% (accelerated). The number of patients with opacifications peaked at 16.5% (accelerated) after 6 months, whereas in the Dresden protocol, it peaked at 12.8% after 3 months. In each group, two patients exhibited infiltrates. A scar developed in eight (Dresden) and four (accelerated) patients. We observed eight (Dresden) and two (accelerated) patients with a persisting epithelial defect, only after 3 months. The rate of oedema peaked in both groups after 3 months, at 16.3% in the Dresden protocol and at 3.8% in the accelerated group.

### Correlation between clinical findings and Pentacam measurements

All patients underwent Pentacam examinations at every clinical visit. Table 2 shows the changes of corneal measurements during therapy. The pre-OP measurements revealed no statistically significant difference between the two groups for Kmax but differences for Kmf and pachymetry were significantly different. To assess significant differences in corneal measurement values in Pentacam examinations between patients with certain findings, the Mann-Whitney U-test was performed.

**Table 2:** Corneal measurements during follow-up period

	pre-OP	3 M	6 M	9 M	12 M	24 M	36 M
Kmax (Dresden) [D]	55.53	55.85	55.32	55.01	55.28	55.43	55.41
Kmax (Accelerated) [D]	54.25	55.34	54.56	54.48	55.33	55.14	52.99
*p*-value	0.06						
KMF (Dresden) [D]	47.76	47.96	47.78	47.41	47.41	48.05	47.94
KMF (Accelerated) [D]	46.34	47.06	46.70	46.66	47.08	47.41	45.80
p-value	<0.01*						
Pachymetry (Dresden) [μm]	457.75	444.92	441.51	445.15	452.18	446.81	443.65
Pachymetry (Acclerated) [μm]	467.62	460.38	455.32	466.17	467.48	462.07	448.38
*p*-value	0.014*						

Corneal measurements during follow-up. *M* months, *Kmax* Maximal K value as measured in keratometry, *Kmf* average keratometry reading of front cornea. *=statistical significant difference

Subsequently, data were compared with the values of a clear cornea. Significant differences between clear corneas and those with striae were seen when comparing Kmax and partly Kmf and pachymetry, but to a greater extent in the accelerated group (Table 4) than in the Dresden protocol group (Table 3).

**Table 3:** Corneal measurements following the Dresden protocol

	Finding	pre-OP	3 M	6 M	9 M	12 M	24 M	36 M
Kmax [D]	clear	53.2	54.6	52.5	53.9	52.8	54.1	53.1
	Striae	56.8	57	54.9	54.5	56.5	55.1	54.8
	p Striae-clear	0*	0.06	0.73	0.64	0.15	0.35	0.01*
	Haze	–	54.9	56.1	52.3	53.2	53.8	50.1
	p Haze-clear	–	0.29	0.43	0.55	0.92	0.78	0.52
Kmf [D]	clear	46.4	47.2	46.1	47.6	47.8	47.3	46.8
	Striae	47.9	48.2	46.9	46.5	46.6	46.9	46.9
	p Striae-clear	0.01*	0.14	0.93	0.49	0.59	0.73	0.13
	Haze	–	47.5	48.2	44.8	45	46.9	45.6
	p Haze-clear	–	0.49	0.58	0.4	0.56	0.95	0.73
Pachymetry [μm]	clear	464.4	454	454	449.5	453	443	459.5
	Striae	453	446.3	439.1	456	450	455	449
	p Striae-clear	0.36	0.25	0.62	0.85	0.84	0.05*	0.49
	Haze	–	448	456.6	457	481	452	439
	p Haze-clear	–	0.5	0.65	0.92	0.44	0.38	0.66
Incidences [number of eyes]	clear	72	37	17	18	17	50	34
	Striae	60	67	59	38	31	52	45
	Haze	0	91	16	6	1	7	1
Eyes		138	129	88	58	53	94	81

Corneal measurements (Dresden protocol, median) obtained during follow-up and concerning corneal findings and incidences. *M* months, *Kmax* maximum K reading in keratometry measurement, *D* dioptres, *Kmf* average keratometry reading of front cornea), *=statistical significant difference

### Predictions and comparison

To determine the likelihood that certain findings changed in the cornea, an analysis with the McNemar test was performed. For the Dresden protocol, significant differences were found concerning the appearance of corneal striae and clarity between pre-surgery corneal findings and during follow-up. The odds ratio for clear corneas was 0.273 after 3 months and 0.152 after 6 months following therapy with the Dresden protocol. For the accelerated protocol, no significant results were obtained. For striae, significantly increased odds ratios were measured in the Dresden protocol treatment group: 4.0 after 6 months and 2.0 after 2 years.

To compare the two treatment protocols for the likelihood of having a clear cornea and of finding haze or striae, an exact Fisher test was performed. For clarity, a significant odds ratio (0.417/0.232) was observed at three and 6 months after treatment. This meant that a clear cornea was more likely to be observed in the accelerated group than in the standard group. In contrast, we measured a significant odds ratio after 3 months (2.073) for striae, and hence striae were more likely to be seen in the Dresden protocol group.

## Discussion

In this study, we investigated and compared changes in corneal clinical findings of keratoconus patients after CXL with the established Dresden protocol and with the accelerated protocol in 286 eyes retrospectively for 3 years following treatment.

During the last few years, several randomized clinical trials have been conducted by using CXL [13–16]. All have shown that keratoconus progression can be halted and, in some cases, keratometry measurements and visual acuity can be improved. However, some controversy regarding the recent Food and Drug Administration (FDA) approval exists [17]. CXL has also proved to be successfully over time, as a long-term stabilization of the cornea has been observed over 10 years [18]. The accelerated CXL treatment is known to be as effective as the standard Dresden protocol [19–23]. The first experimental study was performed in 2011 in Switzerland. In total, 72 porcine eyes were randomly assigned to three different treatment groups. Various CXL illumination intensities were used: at 10 mW/cm^2^ for 9 min and at 3 mW/cm^2^ for 30 min with a constant energy dose of 5.4 J/cm^2^ and a control with no radiation [24]. This investigation revealed that rapid UV CXL treatment can be regarded as equivalent to the standard procedure in terms of the increase in corneal stiffness. However, another study of porcine eyes showed that both the standard and the accelerated protocols increased corneal enzymatic resistance, although the amount of CXL might be less when accelerated CXL is used [25].

As is known from previous studies, corneal CXL induces morphological or inflammatory changes to the cornea, such as haze, but these are resolved mostly after a few months [26, 27]. In addition to clinical changes, other transformations are associated with CXL, such as keratocyte apoptosis and thermomechanical behavioural alterations [28, 29].

Long-term data exists showing corneal CXL to be a safe procedure but, rarely, complications can arise [11, 18]. Most studies so far have focused on measurement data from corneal diagnostics, such as Pentacam or Orbscan examinations [30], in which a differentiation of findings affecting densitometry cannot be made because of the way in which the measurements have been taken. To our knowledge, this is the first study that compares detailed clinical findings of the two protocols over a long period (36 months) in a large cohort.

On first impression (Figs. 1 and 2), the accelerated protocol seems to have fewer effects on clinical findings than the Dresden protocol. Before treatment, a pre-operative haze was noted in three eyes. The average Kmax value of these eyes was 51.2 D before treatment. The two affected patients were lost to follow-up after 9 months. The rate of clear corneas started at the same level with both treatment methods but dropped sharply in the Dresden protocol group. However, after 3 years, both groups returned to a similar level (42% Dresden, 47.6% accelerated), although considerably fewer eyes in the accelerated group could be followed-up for that period (21 vs. 81 eyes). Nonetheless, most of the changes and differences between the two groups were observed in the first 12 months of the study, when both groups had a comparable number of study subjects. For the older protocol, a significant odds ratio of 0.273 was found after 3 months and 0.152 after 6 months, indicating that a baseline clear cornea is again clear after treatment. For the accelerated protocol, no significant difference was determined. The variance could be observed not only graphically, but also by statistical comparison, showing a significant difference after three and 6 months of treatment. The significant odds ratio was 0.417 and 0.232 for three and 6 months, respectively.

For striae, more fluctuation was observed in the patients treated with the conventional protocol. As with clear corneas, the incidence of striae returned to a comparable level after 3 years of follow-up (55.6% Dresden, 52.4% accelerated). However, a larger fluctuation occurred in the Dresden protocol group, as the maximum rate of this feature increased to 67%, compared with a maximum of 52.4% in the accelerated group. For striae, we determined a significant odds ratio of 4.0. after 6 months and 2.0 after 24 months in the Dresden protocol group. The difference in the odds ratio between both groups was significant after 3 months with a value of 2.0.

Obviously, the largest difference occurred for the development of haze. This finding could be observed in 70.5% of patients after conventional treatment and in 46.9% of patients treated by the accelerated protocol. When comparing the two protocols, the odds ratio was 2.0 but did not reach a significant level. The rate of haze in our group is higher than that reported in other publications [30]. This might be because we additionally follow-up our patients for a few days post-operatively and not at longer intervals as in other studies. Furthermore, clinical findings are documented by an experienced observer (WJM) based on subjective grading.

Tables 3 and 4 indicate that corneal measurement values in Pentacam analyses differ between the treatment protocols, when certain clinical findings are present. Differences in measurement values occurred more in the accelerated groups, if one or more corneal findings were present. However, this trend could be observed even pre-operatively. A more progressed keratoconus is known to show striae as a clinical sign. The reason for the detection of a significant difference between clear corneas and those with striae might be that when the accelerated protocol was introduced, more confidence in CXL existed and treatment was initiated earlier. However, no significant difference between the Kmax values of the two treatment groups was seen before surgery.

**Table 4:** Corneal measurements following the accelerated protocol

	Finding	pre-OP	3 M	6 M	9 M	12 M	24 M	36 M
Kmax [D]	clear	51.2	53.06	52.84	53.6	50.85	50.9	50.1
	Striae	56.6	56.6	56.7	56.5	58.7	57.1	57.8
	p Striae-clear	0*	0.03*	0*	0.04*	0*	0*	0*
	Haze	51.6	54.0	49.9	50.6	–	57.9	–
	p Haze-clear	0.83	0.63	0.73	0.71	–	0.23	–
Kmf [D]	clear	44.8	45.5	45.3	45.3	44.8	45.3	44
	Striae	47.1	47.4	48.1	47.3	48.3	48	47.9
	p Striae-clear	0*	0.05*	0.01*	0.15	0*	0*	0*
	Haze	44.3	45.7	45.6	46.1	–	48.1	–
	p Haze-clear	0.39	0.99	0.96	0.94	–	0.29	–
Pachymetry [μm]	clear	472	461.3	461.5	466	480.5	474	453
	Striae	455	451.9	443.5	465	461	457	443
	p Striae-clear	0*	0.16	0.03*	0.59	0.02*	0.05*	0.81
	Haze	483	458.8	440	409	–	479	–
	p Haze-clear	0.84	0.65	0.08	0.2	–	0.79	–
Incidences [number of eyes]	clear	75	61	40	22	20	32	10
	Striae	69	59	44	27	25	29	11
	Haze	3	61	7	1	0	1	0
Eyes		148	130	85	53	49	58	21

Corneal measurements (accelerated treatment, median) obtained during follow-up and concerning. *M* months, *Kmax* maximum K reading in keratometry measurement, *D* dioptres, *Kmf* average keratometry reading of front cornea, corneal findings and incidences, *=statistical significant difference

The nature of this study, namely it being retrospective, is a limitation of this investigation, as are the high numbers of patients who were lost to follow-up. One reason is probably that some patients decided to be followed-up by their local ophthalmologists; this might save them a significant amount of time as most patients are of a working age. The imbalance of patient numbers, in particular towards the end of the study, makes comparisons, especially for the 3-year follow-up, difficult, because 81 eyes are still followed-up in the conventional CXL treatment group and only 21 eyes in the accelerated protocol. This is probably mostly attributable to the novelty of the accelerated protocol, which was introduced in 2013 into our hospital. To show long-term results in the present report, we nevertheless decided to include the three-year figures.

## Conclusion

In conclusion, we can confirm that accelerated CXL is as safe as CXL by using Dresden protocol, despite the observation of fewer changes in corneal clinical findings. As new protocols emerge (e.g. pulsed CXL, topoguided/mosaic-CXL), further studies need to be conducted to evaluate their effects on the cornea.

## Abbreviations

CXL: Cross-linking
D: Dioptres
Kmax: maximal Keratometric measurement value (in dioptres)
Kmf: Keratometric mean measurement front value (in dioptres)
OP: Operation
UVA: Ultraviolet-A
